# Fungal cell wall vaccines: an update

**DOI:** 10.1099/jmm.0.041665-0

**Published:** 2012-07

**Authors:** John E. Edwards

**Affiliations:** Harbor/UCLA Medical Center and Los Angeles Biomedical Research Institute, 1124 West Carson Street, Torrance, CA 90502, USA

## Abstract

This discussion is intended to be an overview of current advances in the development of fungal cell wall vaccines with an emphasis on *Candida*; it is not a comprehensive historical review of all fungal cell wall vaccines. Selected, more recent, innovative strategies for developing fungal vaccines will be highlighted. Both scientific and logistical obstacles related to the development of, and clinical use of, fungal vaccines will be discussed.

## Introduction

In general, over the years there have been extensive research efforts to develop vaccines for both the opportunistic and the endemic fungal infections of man and animals. Several comprehensive reviews and commentaries are available (Deepe, 1997; Perruccio *et al.*, 2004; Feldmesser, 2005; Cutler *et al.*, 2007; Cassone, 2008; Ito *et al.*, 2009; Fidel & Cutler, 2011; Spellberg, 2011). By far, the majority of the work has focused on a vaccine for human infections caused by *Candida* species. However, considerable effort has been extended toward development of vaccines for cryptococcosis, coccidioidomycosis, blastomycosis, histoplasmosis, paracoccidioidomycosis, infections caused by *Pneumocystis* and, more recently, aspergillosis. Despite the extensive investigations of these vaccines, none has yet been approved by the US Food and Drug Administration (FDA) for either active or passive immunization in humans.

Table 1 includes an incomplete listing of various entities explored extensively for development of an active vaccine for haematogenously disseminated and mucosal candidiasis. Not all of these entities and strategies are limited to the cell wall of the organism. Table 2 is an incomplete list of various strategies to enhance the activity of *Candida* vaccines through various adjuvants and delivery systems. Publications describing these strategies have been, in general, highly positive, and have resulted in substantial protection of mice. There is a remarkable paucity of exploration of these vaccines in any other animal models, due at least in part to a greater abundance of knowledge and reagents available relevant to murine immunology. Mice and humans are undoubtedly substantially different regarding their innate immune response to *Candida*. For instance, mice are naïve to the organism, while *Candida* species colonize humans extensively, without causing damage to the host. It may be highly desirable for the preclinical evaluation of fungal vaccines, in general, and specifically for vaccines against *Candida* species, to be conducted in alternative animal models, other than the murine model. While expensive for screening, non-human primates may be a much more suitable model for late-stage vaccine development, since they are not naïve to the organism, and are generally colonized with it, similar to the colonization of humans.

**Table 1.** Various entities explored extensively for development of an active vaccine for haematogenously disseminated and mucosal candidiasis

Various strategies used for active vaccination against *Candida* historically
Heat-killed whole organisms
Attenuated live organisms
*Candida* enolase
*Candida* cell surface iC3b receptors
*Candida* mannans
β-Glucans
Heat-shock protein hsp90
*SAP* gene family proteins
*ALS* gene family proteins
Glycoconjugates (mannans and β-glucans)

**Table 2.** Various strategies to enhance the activity of *Candida* vaccines through various adjuvants and delivery systems

Various strategies to enhance the activity of *Candida* vaccines
Different adjuvants
Nasal delivery systems
Lipid particle delivery systems
DNA delivery
Dendritic cell presentation
Virosome delivery

Notable is that the large body of scientific effort directed towards developing a clinically useful fungal vaccine has not resulted in FDA or other regulatory approval of a single entity thus far, especially with the high level of success found in the preclinical models. This complete absence of approval of a fungal vaccine occurs within the context of an ever increasing frequency of opportunistic fungal infections in susceptible patients, especially related to healthcare-associated infections. Fungal infections occurring in this setting carry a mortality rate of approximately 40–50 %, even in the face of aggressive antifungal drug therapy (Pappas *et al.*, 2003). These healthcare-associated infections are not limited to patients with iatrogenic immunosuppression, organ transplantation or cancer therapy. They also occur in patients who are not treated with immunosuppressive agents, but have been treated with broad-spectrum antibiotics, indwelling intravenous and intra-arterial lines, prosthetic material implants and hyperalimentation fluids, and in patients who are postoperative or are in intensive care units for other reasons. Burn patients and low birth weight neonates are additional populations of susceptible patients.

In this author’s opinion, the reason for the lack of clinically available fungal vaccines is the result of a confluence of issues. First, there is a general under-appreciation for the magnitude of fungal infections for both the opportunistic fungi and the endemic fungi. Second, there are formidable obstacles in ‘technical transfer’ of successful preclinical studies within the field of fungal vaccines. These obstacles include inadequate staffing of offices for technical transfer in many universities, the competitiveness of small business innovative research and small business technical transfer grants for technical transfer, the high cost of preparing antigens for use in human studies through meeting standards for good manufacturing process, the high cost of toxicology studies and the high cost of conducting Phase I clinical trials to establish tolerability and safety. For the development of an antigen through a Phase I clinical trial, the very first stage of testing in humans, approximately 3–4 million dollars (USD) are necessary (personal experience). This is obviously a formidable amount for most academic laboratories to accrue, and, to date, large pharmaceutical companies have not engaged in fungal vaccine development through Phase I trials. However, two biotechnical companies have begun Phase I trials; they will be discussed later.

## Newer innovative strategies

### Live attenuated *Candida* strain

Several highly innovative strategies have been published in recent years and will be selectively highlighted herein. A genetically engineered *Candida albicans* *tet*-*NRG1* strain has been used as an experimental live attenuated vaccine against haematogenously disseminated candidiasis (Saville *et al.*, 2008). This strain is non-pathogenic in the murine model under certain conditions which can be regulated. *NRG1* is a negative regulator of filamentation of *Candida*. In this engineered strain, the gene is under control of a tetracycline-regulatable promoter. In mice not consuming doxycycline in their drinking water, the organism remains in the yeast phase and is non-pathogenic, despite the fact that it can proliferate in tissues. Mice were first infected with this strain as a live attenuated vaccine strategy. A secondary infection with the strain, with the virulence of the organism restored by feeding the mice doxycycline in their drinking water (downregulating *NRG1*, and allowing germination to occur), resulted in substantial protection from the ‘virulence restored’ strain. By using knockout mice, the mechanism of protection was established as being through a T-cell, rather than a B-cell, or neutrophil enhancing mechanism. These results were the highest level of protection described for an anti-*C. albicans* vaccine, especially in immunocompromised mice or mice with neutrophil deficiency. This was a highly innovative and effective active vaccine strategy, and may provide valuable background for further development. However, it does require the use of a live, attenuated strain of *Candida*, which could face formidable challenges regarding safety evaluation by the FDA for approval in humans.

### β-Mannan and peptide conjugates

Another highly innovative approach for *Candida* has been developed by Xin *et al.* (2008). These investigators have created a collection of six glycopeptides vaccines by combining β-mannan and peptide epitopes. Five of the resulting glycopeptides induced protection against murine disseminated candidiasis. The six T-cell peptides from *C. albicans* cell wall proteins were selected by algorithm peptide epitope searches. Each of the peptides was then synthesized and conjugated to the fungal cell wall β-mannan trisaccharide [β-(Man)_3_] by a novel saccharide–peptide linker to create the glycopeptides conjugates. This linker was a non-immunogenic, cross-coupling reagent, which resulted in a stable amide bond. In the immunization strategies, dendritic cell presentation was used, and complete Freund’s adjuvant was used as an adjuvant. Either strong or moderate protection was seen with five of the six glycopeptides. One of the antigens was associated with an increased mortality in challenged control mice compared to challenged vaccinated mice, thereby being non-protective. Of significant importance is that these protective responses were mediated by fully synthetic, chemically defined, immunogens, and represent an approach that may have widespread implications for the development of vaccine antigens. Recent additional studies (Xin & Cutler, 2011) have shown that a peptide alone (Fba) has been highly protective as an active vaccine against several *C. albicans* strains and that the monoclonal antibody specific for the peptide, E2-9 (IgM), was protective when used in passive transfer. These experiments substantiate the role of antibody in protection in this murine model.

### β-Glucan conjugate vaccine for *Candida*

Further exploration of vaccine strategies for *Candida* using cell wall antigens has been accomplished by Pietrella *et al.* (2010). They have used β**-**glucan conjugated with laminarin-CRM together with the adjuvant MF59. Excellent protection was seen for vaginal candidiasis in their murine model. Their studies were of further value in demonstrating the use of a genetically engineered, luminescent *C. albicans* strain, which allowed *in vivo* evaluation of the vaccine efficacy. The *in vivo* evaluation reflected colony counting measurements accurately. The adjuvant MF59 has been used extensively in influenza vaccines in Europe, but it is not currently approved by the FDA for use in the US. It has the property of rapid recruitment and activation of helper CD4^+^ cells. This response is thought to promote higher titres of antibody response. Passive transfer experiments demonstrated the protection in this model to be mediated by antibody. Additionally, passive vaccination with a β-glucan monoclonal antibody resulted in protection against the vaginal candidiasis as well. Of additional interest is that this monoclonal antibody also recognizes the linear epta- or octa-β-1,3 glucan epitope present in the cell wall proteins Als3p and Hyr1p. These proteins are thought to play roles in hyphal growth, and adherence and invasion of human cells. Because of the combined T helper (Th)1 and B-cell activity of MF59, it may be an attractive vaccine adjuvant for fungal vaccines (Brito *et al.*, 2011).

### Sap2p vaccine for vaginitis caused by *Candida* species

This vaccine is based on a recombinant, truncated form of Sap2p. This protein is not actually a cell wall protein, but rather is a secreted aspartyl proteinase. The role of the *SAP* gene family in virulence has been reviewed recently (Correia *et al.*, 2010). The vaccine is targeted to prevent recurrent vulvaginitis. A brief discussion of a clinical trial with this protein is in the later part of this discussion under the human studies section.

### Preclinical studies for the rAls3p-N vaccine

Our group has been working for years on a cell wall protein based-vaccine strategy, which has been recently tested in humans in a Phase I clinical trial (Hennessey *et al.*, 2011). This work began after we discovered that Als1p is an adhesin enabling *Candida* to bind to human vascular endothelial cells. This discovery was made by transforming the non-adherent *Saccharomyces cerevisiae* with genomic *Candida* DNA fragments and testing for conversion of *Saccharomyces* from non-adherent to adherent. The use of surrogate genetics was successful, and cloning of the gene responsible for this conversion led to the identification of *ALS1* as the gene mediating adherence. That discovery occurred shortly after Hoyer *et al.* (1995) had discovered the *ALS* genes in their efforts to determine the genetics responsible for the filamentation of *Candida*. Once the adhesive characteristics of Als1p were determined, we performed classic gene disruption studies, as well as overexpression studies, to confirm that the protein was acting as an adhesin in *C. albicans*. Our research focus split at that time into further investigations of the precise mechanism of adherence, and we began studies to determine whether the protein could act as an immunogen. We produced larger quantities of the protein by recombinant technology in *Saccharomyces cerevisiae*. The protein was purified using a nickel column to precipitate a His-tagged protein encoded by the *C. albicans* gene. Initial vaccination studies included the adjuvant complete Freund’s adjuvant inoculated with the recombinant N-terminus of Als1p (rAls1p-N). For nearly all studies, BALB/c mice were inoculated subcutaneously, boosted in 3 weeks, then infected 2 weeks after the booster with lethal doses of *C. albicans*. Initial studies were performed with the common *C. albicans* strain SC5314. Characteristically, 40–60 % of vaccinated mice survived a 30 day observation time compared to 100 % lethality in 10–14 days in mice receiving adjuvant control. Similar encouraging results were seen in severely neutropenic mice, steroid-treated mice, and in murine models of oropharyngeal candidiasis and *Candida* vaginitis. These studies were advanced into a trial in non-human primates to determine the extent of IgG response when rAls1p-N was given with aluminium hydroxide as the adjuvant. IgG titres rose by 1–2 logs in these primates, which, unlike mice, are not naïve to *Candida*.

Since Phan *et al.* (2007) showed that Als3p was not only an adhesin, but also an invasin for *Candida* in endothelial cells, rAls3p-N was tested as a vaccine and was shown to be approximately equal in efficacy to rAls1p-N. Because of this dual property of Als3p, our studies were continued using it as the primary antigen. The combination of rAslp-N and rAls3p-N did not show increased efficacy over either antigen alone.

Adoptive transfer experiments using B-cell and T-cell knockout mice were used to explore the mechanism of the vaccine protection. B-cell knockout mice transfused with B-cell lymphocytes harvested from mice, which had been vaccinated with both rAls1p-N and rAls3p-N, did not show protection when challenged with *C. albicans*. Also, serum from mice vaccinated with rAls3p-N was not protective in the lethal challenge model. Furthermore, IFN-γ-deficient mice were not protected by the vaccine. However, in T-cell knockout mice which were transfused with T cells harvested from vaccinated mice, protection against the lethal challenge was observed, demonstrating that the major mechanism of protection was due primarily to T cells and not due to B cells or antibody production by the vaccine (Ibrahim *et al.*, 2005). However, antibody production was induced in the mice by the vaccine.

These studies have been extended further to define the mechanism of protection (Lin *et al.*, 2009). In these experiments, neither mice with cyclophosphamide-induced neutropenia nor mice deficient in gp91*^phox−/−^* (mice which have neutrophils unable to generate superoxide and have neutrophil microbial killing deficiency) were protected by vaccination with rAls3p-N, indicating that functional neutrophils were essential for the vaccine mechanism of protection. Adoptive transfer of CD4^+^ positive lymphocytes from vaccinated mice into the gp91*^phox−/−^* mice also failed to protect, as anticipated.

To determine whether IL-17 was necessary for the vaccine mechanism of action, mice deficient in IL-17A were vaccinated and compared to wild-type vaccinated control mice. No vaccine-induced protection was seen in the IL-17A-deficient mice in comparison to the vaccinated and protected control mice, suggesting that, in part, the vaccine required IL-17A. When unvaccinated IL-17A-deficient mice were compared to unvaccinated control mice and challenged with *C. albicans*, no difference was seen, suggesting that IL-17A is not necessary for innate protection, not related to vaccination.

To determine whether CD4^+^ cells were the source of the IL-17A, IL-17A-deficient donor CD4^+^ cells were transferred to wild-type recipient mice and wild-type CD4^+^ donor cells were transferred to IL-17A-deficient recipient mice. The CD4^+^ cells from vaccinated wild-type mice resulted in protection of the IL-17A-deficient mice challenged with *C. albicans*. As anticipated, transfer of CD4^+^ cells from vaccinated IL-17A-deficient mice did not protect against the *C. albicans* challenge. These experiments are evidence that CD4^+^ cells are the source of IL-17A in vaccinated mice.

To define the populations of cells induced by vaccination, spleens and lymph nodes were taken from vaccinated and control non-vaccinated mice 2 weeks after the boost of the vaccinated mice. These cells were stimulated with rAls3p-N. IFN-γ and IL-17, as well as neutrophil activating chemokines KC and MIP-1α, were produced in higher quantity than in the non-vaccinated control mice cells. Supernatants from the vaccinated mice stimulated neutrophils much more extensively than supernatants from the non-vaccinated control mice. Additionally, vaccinated mice had a substantial reduction in kidney fungal burden; myeloperoxidase levels were increased in the kidneys in vaccinated mice, as well as the influx of neutrophils, as quantified on histopathology. Additionally, increased levels of IFN-γ, IL-17 and the neutrophil activating cytokine CXC were found in the kidneys.

In vaccinated mice, there were higher levels of Th1, Th17 and Th1/17 cells than in non-vaccinated controls. CD4^+^CCR6^−^ cells were enriched in the Th1 cells; CD4^+^CCR6^+^ cells were enriched in the Th17 cells. A substantial portion of the spleen and lymph node CD4^+^CCR6^+^ cells were Th1/17 cells.

In summary regarding the mechanism of action of the rAls3p-N vaccine in mice, the following points can be made. IFN-γ and IL-17A were necessary for vaccine-induced protection in mice. The source of the IL-17A and IFN-γ was Th1, Th17 and Th1/17 lymphocytes. These cytokines mediated recruitment of neutrophils, due to CXC and MIP-1α, to the site of infection, which resulted in a decreased tissue burden. IL-17A was not necessary for defence against *C. albicans* challenge in non-vaccine immune defence, but was necessary in vaccine-induced protection.

## Protection against *Staphylococcus* by the *Candida* rAls3p-N vaccine

In our studies of the molecular mechanism of attachment of *C. albicans* to human cells, we performed molecular modelling of all the Als proteins (Sheppard *et al.*, 2004). Of great interest is that high levels, 80–90 %, of three-dimensional (3D) homology were found between the Als proteins and cell surface molecules on non-fungal organisms, including bacteria and viruses. Of note was the homology found with clumping factor (ClfA), a cell surface molecule of *Staphylococcus aureus*. When identifying this 3D homology, we decided to determine whether rAls3p-N could induce a protective response as a vaccine against *S. aureus*. Mice were immunized with rAls3p-N, according to standard protocols used for the *C. albicans* vaccination, and then challenged with *S. aureus*. Protection results were similar to those for *C. albicans*, demonstrating a ‘cross-kingdom’ protection (Spellberg *et al.*, 2008). Extensive studies of the mechanism of the protection have shown it to be essentially identical to the mechanism for rAls3p-N protection, primarily a T-cell mechanism rather than a B-cell mechanism and not dependent primarily on antibody, as determined by adoptive transfer experiments.

## Current human studies of vaccines against *Candida* species

### Human studies of the rAls3p-N *Candida* vaccine

These studies described above have led to a Phase I clinical trial in human subjects performed by NovaDigm Therapeutics (Hennessey *et al.*, 2011). Subjects (30) were given two doses of vaccine, 30 µg and 300 µg. The vaccine induced no related adverse events and was well tolerated. It should be noted that all the studies of the mechanism of action of the vaccine to date have been done in the murine model and may not translate perfectly to humans. For instance, the Phase I clinical trial in humans showed a robust production of IgG and IgA1 antibodies in the majority of the subjects (Hennessey *et al.*, 2011). Most of the human subjects also had an increase in production of IFN-γ as well as IL-17A, as determined by ELISpot measurement. These observations suggest that in humans, the T cells, B cells and antibodies are all stimulated by the vaccine. We have retained peripheral blood mononuclear cells (PBMCs) and serum from the human subjects in their pre- and post-vaccinated state, and now have the capability of defining, in much more detail, the human vaccine response to this purified fungal antigen. Of great interest will be determining the opsonophagocytic activity of the human serum for both *Candida* and *Staphylococcus.* These studies are currently in progress. Also a detailed study of the T-cell response is planned for the PBMCs. Studying these human pre- and post-vaccine resources should allow a highly enhanced definition of the mechanism of action of the vaccine, as well as possibly defining more useful surrogate markers, or combination of markers, for determining vaccine efficacy in future trials.

If this vaccine proves to be efficacious, it could have utility in a variety of clinical settings including vaginitis caused by *Candida* species, skin and soft tissue infections caused by *S. aureus*, and in patients who acquire either organism in the healthcare-associated setting, particularly in the intensive care unit. In the latter circumstance, the vaccine would be given to selected patients who are in high-risk groups, such as patients undergoing cardiovascular, thoracic or gastrointestinal surgery. Many of these patients could receive the vaccine preoperatively, even before entry to the intensive care unit. Patients with immunosuppression (iatrogenic, such as those treated with cytotoxic cancer chemotherapy) are far less likely to benefit from an active vaccine, and do not comprise the largest group of patients acquiring bacteraemia or other infections caused by these two organisms. Such patients may benefit from a passive vaccine given either alone or in combination with an active vaccine. Obviously, it would be highly desirable for an active vaccine to have a rapid onset of efficacy, when used in this healthcare-associated setting. In that context, the rAls3p-N vaccine elicited high antibody titres within 3–7 days after administration in the completed Phase I clinical trial (Hennessey *et al.*, 2011). Unlike vaccines given to large populations of subjects designed to create ‘herd’ immunity, the duration of efficacy for a vaccine used to prevent or ameliorate healthcare-associated infections would need to be efficacious only during the time the subjects were at risk for infection.

### Human trials of the Sap2p vaccine for vaginitis caused by *Candida* species

In 2010, a clinical trial based on a recombinant, truncated form of Sap2p was begun in Europe. The vaccine is targeted to prevent recurrent vulvaginitis. It is being delivered in both intramuscular and intravaginal forms with a proprietary virosome. This active vaccine is being developed by Pevion Biotech, and interim data indicate that it is well tolerated and is ‘effective’ at low doses. A full description of the results of the Phase I trial is not currently in the public domain.

## Vaccine development for other fungal pathogens causing haematogenously disseminated disease

### Coccidioidomycosis

Currently, vaccine development for other fungal organisms has not reached clinical trials, with the exception of vaccines for *Coccidioidomyces*. A clinical trial for a vaccine for coccidioidomycosis was performed as long ago as 1975 (Pappagianis & Levine, 1975), and again in 1993 (Pappagianis & The Valley Fever Vaccine Study Group, 1993). However, there has not been further advancement of trials in humans for over 18 years now, and a vaccine for the prevention of coccidioidomycosis has not yet been approved by the FDA. However, extensive preclinical development of a coccidioidomycosis vaccine is still under way (Shubitz *et al.*, 2008; Capilla *et al.*, 2009; Xue *et al.*, 2009). Previous studies have included a variety of strategies. For instance, a recombinant enzyme antigen, a 4-dehydroxyphenylpyruvate dioxygenase, has been shown to be protective in mice against an intraperitoneal challenge with arthroconidia of *Coccidioides* (Wyckoff *et al.*, 1995). Additionally, an extract of the outer cell wall of spherules (SOW) has also conveyed protection in the same mouse model (Wyckoff *et al.*, 1995). Further studies with a water-soluble antigen (C-ASWS) showed partial protection in mice with a natural relative resistance to *Coccidioides immitis* (Kirkland & Fierer, 1985). Additional studies have been performed with a 33 kDa antigen isolated from spherules (Galgiani *et al.*, 1996). This antigen was recognized from sera of patients who had recovered from natural infection. Other more recent studies have included a variety of other antigens and approaches (Kirkland *et al.*, 2006; Tarcha *et al.*, 2006; Awasthi, 2007; Johnson *et al.*, 2007a, b; Lunetta *et al.*, 2007).

Of interest is an important recent study that examined the mechanism of action of a live, attenuated mutant of *Coccidioides posadasii*, used as a vaccine in mice. The attenuated organism has a double gene knockout, *CTS2* and *CTS3*, which are both chitinase genes (Xue *et al.*, 2009). This attenuated mutant is not able to sporulate. Mice are fully protected against pulmonary coccidioidomycosis by this vaccine. Profiles of the cytokines found in lung homogenates of the vaccinated mice, which were challenged with intranasal organisms, showed a mixed Th1-, 2- and 17-type immune response. Functional Th17 cells were necessary for protection by the vaccine in this model, adding to the growing evidence for the role of Th17 cells in the mechanism of protection of most of the fungal vaccines under evaluation (Hung *et al.*, 2011). If this strategy is to move forward into human trials, concerns over the potential for this organism, a eukaryote, to mutate back to a virulent form will require addressing. However, there are several live, attenuated viruses in clinical use today.

### Blastomycosis

Historically, considerable effort has been made by the group in Wisconsin, led by Bruce Klein, to both elucidate the mechanisms of natural resistance to *Blastomyces dermatitidis*, and to develop a vaccine for blastomycosis. A highly immunogenic cell wall protein, WI-1 (now designated BAD-1), has been the major focus (Klein & Jones, 1994). This antigen stimulates humoral and T-cell immunity as well as serving as a ligand for phagocytes. This antigen has been protective in mice, probably mainly through a Th2-type response. Recently, the group has used the innovative strategy of a live, attenuated vaccine in beagles and foxhounds (Wüthrich *et al.*, 2011a). BAD-1 is a cell surface protein, which is an adhesion for *B. dermatitidis* (Brandhorst *et al.*, 1999). Additionally, it has also been shown to be essential for pathogenicity in the mouse model for lethal pulmonary infection. A strain of *B. dermatitidis* has been mutated through knocking out the gene encoding BAD-1 and given live to beagles and foxhounds subcutaneously, without adjuvant. The safety of this vaccine was established; it was also immunogenic. A protection evaluation was not part of this initial experimental design for developing a canine vaccine for blastomycosis.

In this study, lymph nodes were reactive, showing an increase in well-differentiated lymphocytes and follicules, as well as macrophages, and the presence of vaccine organisms. No evidence of dissemination of the attenuated vaccine organisms was found in the lung samples. *In vitro* lymphocyte proliferation was induced with a protective cell wall antigen from *B. dermatitidis*. Increased transcript levels of IFN-γ and TNF-α were found, as well as of GM-CSF, but not of IL-4. The immune responses in this study were vaccine dose-dependent. In general, cellular immunity has been found to be operative in vaccine responses for blastomycosis (Deepe *et al.*, 2005).

Of considerable interest is recent preclinical work on the mechanism of vaccine-induced protection against three systemic mycoses endemic to North America (*Coccidioides* *posadasii*, *Histoplasma capsulatum* and *B. dermatitidis*) (Wüthrich *et al.*, 2011b). The role of Th17 cells in natural innate immunity for fungal infections has been controversial, and has differed somewhat from fungal organism to fungal organism. In these elegant studies, knockout mice and adoptive transfer technology were used extensively. CD4^+^ cells from draining nodes produced IL-17 and IFN-γ in response to antigen and were antigen-specific. To determine whether primed IL-17 cells migrated to the lung of vaccinated mice after pulmonary challenge, transcript and the number of cytokine-producing lung lymphocytes were assayed. Th17 cells were necessary and sufficient for protection against these three organisms. Furthermore, protection was dependent upon Myd88, but not Dectin-1. IL-17A, IL-17F, IL-22 and IFN-γ were elevated. Of interest is that these findings differ from those of one report, in which vaccinated mice with Th17 polarization had increased inflammation of both gastric candidiasis and pulmonary aspergillosis (Zelante *et al.*, 2007). However, in the mouse model for haematogenously disseminated candidiasis, Th17 polarization has been essential for vaccine-induced protection (Lin *et al.*, 2009). These studies strongly suggest that human vaccines for systemic fungal infections should be designed to induce Th17 cells as well as antibodies.

### Histoplasmosis

Historically, efforts have been made to develop a vaccine for the prevention of histoplasmosis. Extracts of the cell wall and cell membrane (CW/M) (Garcia & Howard, 1971), as well as a ribosomal–protein complex, have been evaluated with successful results (Tewari *et al.*, 1978). A protein subfraction of the CW/M preparation, termed HIS-62, was found to be efficacious in three different mouse models (Gomez *et al.*, 1995). HIS-80, another CW/M derivative, has also been protective in mouse models (Gomez *et al.*, 1991). More recently, a heat-shock protein has shown protection in mice (Scheckelhoff & Deepe, 2002). Another innovative approach taken recently is the use of apoptotic neutrophils (Hsieh *et al.*, 2011).

### Aspergillosis

Attention has also been directed toward vaccine strategies for *Aspergillus* (Ito *et al.*, 2009). This organism is particularly problematic in haematopoietic cell transplantation (HCT) recipients, who have response rates to conventional therapy of approximately 31 % (Herbrecht *et al.*, 2002). L. Romani and the mycology group at the University of Perugia, Italy, over a decade ago, demonstrated that they could protect neutropenic mice with a crude culture filtrate (CCFA) vaccine given intranasally prior to induction of neutropenia. Mice were challenged with intranasal inhalation of *Aspergillus* spores. Later they also demonstrated that the allergen Asp f 16, when given with oligodeoxynucleotide adjuvants, was also protective (Bozza *et al.*, 2002). They have also shown that dendritic cells pulsed with *Aspergillus* conidia, or transfected with conidial RNA, were protective for mice undergoing HCT (Bozza *et al.*, 2003). Additionally, dendritic cells transduced with an adenoviral vector encoding production of IL-12 and pulsed with *Aspergillus* has been effective in mice (Shao *et al.*, 2005). Furthermore, a glucan from *Laminaria digitata* conjugated with the diphtheria toxoid CRM197 has been effective for protecting against an intravenous challenge with *Aspergillus* in mice (Torosantucci *et al.*, 2005).

J. Ito and the group at City of Hope have attempted vaccination in mice with freeze–thawed hyphae and culture supernatants of *Aspergillus*. After efficacy was established, they identified the allergen Asp f 3 as the likely immunogen (Ito *et al.*, 2009). After synthesizing recombinant Asp f 3, they were able to establish protection in mice with the protein in combination with the adjuvant TiterMax. Since Asp f 3 is an allergen, and to avoid the possibility of inducing an allergic response, truncated forms of the protein were used, which were also protective in steroid-suppressed mice. They have further defined the protective epitopes of the truncated proteins that were responsible for T-cell Asp f 3 specific stimulation (Ito *et al.*, 2009).

### Pneumocystis, paracoccidioidomycosis and cryptococcosis

A historical review of vaccine development for pneumocystis, paracoccidioidomycosis and cryptococcosis is beyond the scope of this update. Extensive efforts have been made for all three diseases. Encouraging results have been seen recently with a DNA vaccine strategy for pneumocystis (Feng *et al.*, 2011) and paracoccidioidomycosis (Fernandes *et al.*, 2011). A recent study has been published regarding active vaccination for cryptococcosis (Chow & Casadevall, 2011). In this study, a galactoxylomannan–protein conjugate was used in mice. Although it did stimulate antibody production, it failed to result in protection of vaccinated mice compared to controls.

### Recent innovative studies with *Saccharomyces*

Of further interest is the work performed by the group at the Santa Clara Valley Medical Center affiliated with Stanford University, led by David Stevens. It represents a return to earlier studies inducing immunity in mice to *Candida* by vaccination with heat-killed organisms. The group has shown protection in mice by vaccination with heat-killed *Saccharomyces cerevisiae* against *Aspergillus*, *Coccidioides*, *Cryptococcus* and *Candida* (Capilla *et al.*, 2009; Liu *et al.*, 2011a, b; Stevens *et al.*, 2011). The killed *Saccharomyces* induced a specific and comprehensive immune response; both Th1 and Th2. CD4^+^ and CD8^+^ cells were stimulated as well as antibody against *Saccharomyces* glucan and mannan. IFN-γ, IL-6 and IL-17A cytokine production was stimulated also. Alum enhanced the vaccine effect. The constituents in the heat-killed preparation inducing the protective response are not currently known. The IL-17A production is consistent with the growing body of literature verifying that the Th17 pathway is important, not only for innate protection against most fungal organisms, but also for efficacious vaccine responses.

## Summary

In summary, for decades a variety of cell wall antigens (and non-cell wall antigens) from both the opportunistic and the indigenous fungi have been evaluated in preclinical studies. Nearly all of this work has been done in mouse models of vaccination followed by challenge of the fungal organism. There is an extreme paucity of data in humans, due to the small number of clinical trials which have been done to date.

With the exception of the early trials with preparations from *Coccidioides immitis*, at a time when only limited data were derived from the blood of subjects, none of the preclinical work has translated into human trials until recently, when two Phase I trials in human subjects started. Both trials are limited to strategies for prevention of candidiasis. A major reason for the lack of human trials for fungal vaccines has been the very high costs related to good manufacturing process production of the antigens, toxicity studies in animals, and high costs for clinical trials in humans. At present, approximately 3–4 million dollars (USD) are necessary for these steps to be completed. This sum does not include the cost of any clinical trials beyond Phase I. Of encouragement is the growing interest in the private sector of participating in the development of fungal vaccines, especially to address the ever growing problem of healthcare-associated infections. It is likely that in most countries these large costs will be covered by either private funding sources or a combination of private and governmental funds, rather than governmental funds alone.

As data emerge from these early clinical studies, the usefulness of the mouse model for the preclinical development of fungal vaccines will become substantially more clear. However, it is highly likely that the mouse model will have limited utility in accurately predicting vaccine efficacy in humans. If that situation is established, other models will be explored for their utility in preclinical exploration for vaccine strategies not only to prevent fungal infections but to have the added advantage of slowing resistance development to the currently highly limited fungal armamentarium.

## References

[r1] Awasthi S. **(**2007**).** Dendritic cell-based vaccine against coccidioides infection. Ann N Y Acad Sci 1111, 269–274 10.1196/annals.1406.013 17363430

[r2] Bozza S. Gaziano R. Lipford G. B. Montagnoli C. Bacci A. Di Francesco P. Kurup V. P. Wagner H. Romani L. **(**2002**).** Vaccination of mice against invasive aspergillosis with recombinant *Aspergillus* proteins and CpG oligodeoxynucleotides as adjuvants. Microbes Infect 4, 1281–1290 10.1016/S1286-4579(02)00007-2 12443892

[r3] Bozza S. Perruccio K. Montagnoli C. Gaziano R. Bellocchio S. Burchielli E. Nkwanyuo G. Pitzurra L. Velardi A. Romani L. **(**2003**).** A dendritic cell vaccine against invasive aspergillosis in allogeneic hematopoietic transplantation. Blood 102, 3807–3814 10.1182/blood-2003-03-0748 12791648

[r4] Brandhorst T. T. Wüthrich M. Warner T. Klein B. **(**1999**).** Targeted gene disruption reveals an adhesin indispensable for pathogenicity of *Blastomyces dermatitidis*. J Exp Med 189, 1207–1216 10.1084/jem.189.8.1207 10209038 PMC2193031

[r5] Brito L. A. Chan M. Baudner B. Gallorini S. Santos G. O’Hagan D. T. Singh M. **(**2011**).** An alternative renewable source of squalene for use in emulsion adjuvants. Vaccine 29, 6262–6268 10.1016/j.vaccine.2011.06.067 21723355

[r6] Capilla J. Clemons K. V. Liu M. Levine H. B. Stevens D. A. **(**2009**).** *Saccharomyces cerevisiae* as a vaccine against coccidioidomycosis. Vaccine 27, 3662–3668 10.1016/j.vaccine.2009.03.030 19464548

[r7] Cassone A. **(**2008**).** Fungal vaccines: real progress from real challenges. Lancet Infect Dis 8, 114–124 10.1016/S1473-3099(08)70016-1 18222162

[r8] Chow S. K. Casadevall A. **(**2011**).** Evaluation of *Cryptococcus neoformans* galactoxylomannan-protein conjugate as vaccine candidate against murine cryptococcosis. Vaccine 29, 1891–1898 10.1016/j.vaccine.2010.12.134 21238568 PMC3043149

[r9] Correia A. Lermann U. Teixeira L. Cerca F. Botelho S. da Costa R. M. G. Sampaio P. Gärtner F. Morschhäuser J. **& other authors (**2010**).** Limited role of secreted aspartyl proteinases Sap1 to Sap6 in *Candida albicans* virulence and host immune response in murine hematogenously disseminated candidiasis. Infect Immun 78, 4839–4849 10.1128/IAI.00248-10 20679440 PMC2976357

[r10] Cutler J. E. Deepe G. S. Jr Klein B. S. **(**2007**).** Advances in combating fungal diseases: vaccines on the threshold. Nat Rev Microbiol 5, 13–28 10.1038/nrmicro1537 17160002 PMC2214303

[r11] Deepe G. S. Jr **(**1997**).** Prospects for the development of fungal vaccines. Clin Microbiol Rev 10, 585–596 9336663 10.1128/cmr.10.4.585 PMC172935

[r12] Deepe G. S. Jr Wüthrich M. Klein B. S. **(**2005**).** Progress in vaccination for histoplasmosis and blastomycosis: coping with cellular immunity. Med Mycol 43, 381–389 10.1080/13693780500245875 16178365

[r13] Feldmesser M. **(**2005**).** Prospects of vaccines for invasive aspergillosis. Med Mycol 43, 571–587 10.1080/13693780500402138 16396243

[r14] Feng Y. Guo S. Jiang T. Han X. Liu P. Wu T. Luo Y. **(**2011**).** Active immunization against *Pneumocystis carinii* with p55-v3 DNA vaccine in rats. Can J Microbiol 57, 375–381 10.1139/w11-023 21529125

[r15] Fernandes V. C. Martins E. M. Boeloni J. N. Coitinho J. B. Serakides R. Goes A. M. **(**2011**).** Additive effect of rPb27 immunization and chemotherapy in experimental paracoccidioidomycosis. PLoS ONE 6, e17885 10.1371/journal.pone.0017885 21423771 PMC3053394

[r16] Fidel P. L. Jr Cutler J. E. **(**2011**).** Prospects for development of a vaccine to prevent and control vaginal candidiasis. Curr Infect Dis Rep 13, 102–107 10.1007/s11908-010-0143-y 21308461 PMC3062364

[r17] Galgiani J. N. Peng T. Lewis M. L. Cloud G. A. Pappagianis D. The National Institute of Allergy and Infectious Diseases Mycoses Study Group **(**1996**).** Cerebrospinal fluid antibodies detected by ELISA against a 33-kDa antigen from spherules of *Coccidioides immitis* in patients with coccidioidal meningitis. J Infect Dis 173, 499–502 10.1093/infdis/173.2.499 8568322

[r18] Garcia J. P. Howard D. H. **(**1971**).** Characterization of antigens from the yeast phase of *Histoplasma capsulatum*. Infect Immun 4, 116–125 5005290 10.1128/iai.4.2.116-125.1971 PMC416274

[r19] Gomez F. J. Gomez A. M. Deepe G. S. Jr **(**1991**).** Protective efficacy of a 62-kilodalton antigen, HIS-62, from the cell wall and cell membrane of *Histoplasma capsulatum* yeast cells. Infect Immun 59, 4459–4464 1937804 10.1128/iai.59.12.4459-4464.1991 PMC259063

[r20] Gomez F. J. Allendoerfer R. Deepe G. S. Jr **(**1995**).** Vaccination with recombinant heat shock protein 60 from *Histoplasma capsulatum* protects mice against pulmonary histoplasmosis. Infect Immun 63, 2587–2595 7790073 10.1128/iai.63.7.2587-2595.1995 PMC173347

[r21] Hennessey J. P. Jr Schmidt C. S. Ibrahim A. Filler S. White C. J. Yeaman M. Fu Y. Edwards J. E. **(**2011**).** A Phase 1 clinical evaluation of NDV3, a vaccine to prevent disease caused by *Candida* spp. and *Staphylococcus aureus*. In 51st Interscience Conference on Antimicrobial Agents and Chemotherapy, Chicago. Washington, DC: American Society for Microbiology

[r22] Herbrecht R. Denning D. W. Patterson T. F. Bennett J. E. Greene R. E. Oestmann J. W. Kern W. V. Marr K. A. Ribaud P. **& other authors (**2002**).** Voriconazole versus amphotericin B for primary therapy of invasive aspergillosis. N Engl J Med 347, 408–415 10.1056/NEJMoa020191 12167683

[r23] Hoyer L. L. Scherer S. Shatzman A. R. Livi G. P. **(**1995**).** *Candida albicans* ALS1: domains related to a *Saccharomyces cerevisiae* sexual agglutinin separated by a repeating motif. Mol Microbiol 15, 39–54 10.1111/j.1365-2958.1995.tb02219.x 7752895

[r24] Hsieh S. H. Lin J. S. Huang J. H. Wu S. Y. Chu C. L. Kung J. T. Wu-Hsieh B. A. **(**2011**).** Immunization with apoptotic phagocytes containing *Histoplasma capsulatum* activates functional CD8^+^ T cells to protect against histoplasmosis. Infect Immun 79, 4493–4502 10.1128/IAI.05350-11 21911464 PMC3257918

[r25] Hung C. Y. Gonzalez A. Wüthrich M. Klein B. S. Cole G. T. **(**2011**).** Vaccine immunity to coccidioidomycosis occurs by early activation of three signal pathways of T helper cell response (Th1, Th2, and Th17). Infect Immun 79, 4511–4522 10.1128/IAI.05726-11 21859851 PMC3257932

[r26] Ibrahim A. S. Spellberg B. J. Avenissian V. Fu Y. Filler S. G. Edwards J. E. Jr **(**2005**).** Vaccination with recombinant N-terminal domain of Als1p improves survival during murine disseminated candidiasis by enhancing cell-mediated, not humoral, immunity. Infect Immun 73, 999–1005 10.1128/IAI.73.2.999-1005.2005 15664943 PMC547099

[r27] Ito J. I. Lyons J. M. Diaz-Arevalo D. Hong T. B. Kalkum M. **(**2009**).** Vaccine progress. Med Mycol 47 (Suppl. 1), S394–S400 10.1080/13693780802552614 19247869

[r28] Johnson S. M. Kerekes K. M. Lunetta J. M. Pappagianis D. **(**2007a**).** Characteristics of the protective subcellular coccidioidal T27K vaccine. Ann N Y Acad Sci 1111, 275–289 10.1196/annals.1406.016 17363436

[r29] Johnson S. M. Lerche N. W. Pappagianis D. Yee J. L. Galgiani J. N. Hector R. F. **(**2007b**).** Safety, antigenicity, and efficacy of a recombinant coccidioidomycosis vaccine in cynomolgus macaques (Macaca fascicularis). Ann N Y Acad Sci 1111, 290–300 10.1196/annals.1406.042 17347333

[r30] Kirkland T. N. Fierer J. **(**1985**).** Genetic control of resistance to *Coccidioides immitis*: a single gene that is expressed in spleen cells determines resistance. J Immunol 135, 548–552 3998472

[r31] Kirkland T. N. Raz E. Datta S. K. **(**2006**).** Molecular and cellular mechanisms of protective immunity to coccidioidomycosis. Vaccine 24, 495–500 10.1016/j.vaccine.2005.07.092 16181709

[r32] Klein B. S. Jones J. M. **(**1994**).** Purification and characterization of the major antigen WI-1 from *Blastomyces dermatitidis* yeasts and immunological comparison with A antigen. Infect Immun 62, 3890–3900 7520423 10.1128/iai.62.9.3890-3900.1994 PMC303045

[r33] Lin L. Ibrahim A. S. Xu X. Farber J. M. Avanesian V. Baquir B. Fu Y. French S. W. Edwards J. E. Jr Spellberg B. **(**2009**).** Th1-Th17 cells mediate protective adaptive immunity against *Staphylococcus aureus* and *Candida albicans* infection in mice. PLoS Pathog 5, e1000703 10.1371/journal.ppat.1000703 20041174 PMC2792038

[r34] Liu M. Clemons K. V. Bigos M. Medovarska I. Brummer E. Stevens D. A. **(**2011a**).** Immune responses induced by heat killed *Saccharomyces cerevisiae*: a vaccine against fungal infection. Vaccine 29, 1745–1753 10.1016/j.vaccine.2010.12.119 21219976 PMC5508752

[r35] Liu M. Capilla J. Johansen M. E. Alvarado D. Martinez M. Chen V. Clemons K. V. Stevens D. A. **(**2011b**).** *Saccharomyces* as a vaccine against systemic aspergillosis: ‘*the friend of man*’ a friend again? J Med Microbiol 60, 1423–1432 10.1099/jmm.0.033290-0 21825307

[r36] Lunetta J. M. Simmons K. A. Johnson S. M. Pappagianis D. **(**2007**).** Molecular cloning and expression of a cDNA encoding a *Coccidioides posadasii* 1,2-alpha-mannosidase identified in the coccidioidal T27K vaccine by immunoproteomic methods. Ann N Y Acad Sci 1111, 164–180 10.1196/annals.1406.015 17363438

[r37] Pappagianis D. Levine H. B. **(**1975**).** The present status of vaccination against coccidioidomycosis in man. Am J Epidemiol 102, 30–41 1155435 10.1093/oxfordjournals.aje.a112131

[r38] Pappagianis D. The Valley Fever Vaccine Study Group **(**1993**).** Evaluation of the protective efficacy of the killed *Coccidioides immitis* spherule vaccine in humans. Am Rev Respir Dis 148, 656–660 10.1164/ajrccm/148.3.656 8368636

[r39] Pappas P. G. Rex J. H. Lee J. Hamill R. J. Larsen R. A. Powderly W. Kauffman C. A. Hyslop N. Mangino J. E. **& other authors (**2003**).** A prospective observational study of candidemia: epidemiology, therapy, and influences on mortality in hospitalized adult and pediatric patients. Clin Infect Dis 37, 634–643 10.1086/376906 12942393

[r40] Perruccio K. Bozza S. Montagnoli C. Bellocchio S. Aversa F. Martelli M. Bistoni F. Velardi A. Romani L. **(**2004**).** Prospects for dendritic cell vaccination against fungal infections in hematopoietic transplantation. Blood Cells Mol Dis 33, 248–255 10.1016/j.bcmd.2004.08.011 15528139

[r41] Phan Q. T. Myers C. L. Fu Y. Sheppard D. C. Yeaman M. R. Welch W. H. Ibrahim A. S. Edwards J. E. Jr Filler S. G. **(**2007**).** Als3 is a *Candida albicans* invasin that binds to cadherins and induces endocytosis by host cells. PLoS Biol 5, e64 10.1371/journal.pbio.0050064 17311474 PMC1802757

[r42] Pietrella D. Rachini A. Torosantucci A. Chiani P. Brown A. J. Bistoni F. Costantino P. Mosci P. d’Enfert C. **& other authors (**2010**).** A β-glucan-conjugate vaccine and anti-β-glucan antibodies are effective against murine vaginal candidiasis as assessed by a novel in vivo imaging technique. Vaccine 28, 1717–1725 10.1016/j.vaccine.2009.12.021 20038431

[r43] Saville S. P. Lazzell A. L. Chaturvedi A. K. Monteagudo C. Lopez-Ribot J. L. **(**2008**).** Use of a genetically engineered strain to evaluate the pathogenic potential of yeast cell and filamentous forms during *Candida albicans* systemic infection in immunodeficient mice. Infect Immun 76, 97–102 10.1128/IAI.00982-07 17967861 PMC2223671

[r44] Scheckelhoff M. Deepe G. S. Jr **(**2002**).** The protective immune response to heat shock protein 60 of *Histoplasma capsulatum* is mediated by a subset of Vβ8.1/8.2^+^ T cells. J Immunol 169, 5818–5826 12421963 10.4049/jimmunol.169.10.5818

[r45] Shao C. Qu J. He L. Zhang Y. Wang J. Zhou H. Wang Y. Liu X. **(**2005**).** Dendritic cells transduced with an adenovirus vector encoding interleukin-12 are a potent vaccine for invasive pulmonary aspergillosis. Genes Immun 6, 103–114 10.1038/sj.gene.6364167 15674391

[r46] Sheppard D. C. Yeaman M. R. Welch W. H. Phan Q. T. Fu Y. Ibrahim A. S. Filler S. G. Zhang M. Waring A. J. Edwards J. E. Jr **(**2004**).** Functional and structural diversity in the Als protein family of *Candida albicans*. J Biol Chem 279, 30480–30489 10.1074/jbc.M401929200 15128742

[r47] Shubitz L. F. Dial S. M. Perrill R. Casement R. Galgiani J. N. **(**2008**).** Vaccine-induced cellular immune responses differ from innate responses in susceptible and resistant strains of mice infected with *Coccidioides posadasii*. Infect Immun 76, 5553–5564 10.1128/IAI.00885-08 18852250 PMC2583549

[r48] Spellberg B. **(**2011**).** Vaccines for invasive fungal infections. F1000 Med Rep 3, 13 10.3410/M3-13 21876719 PMC3155210

[r49] Spellberg B. Ibrahim A. S. Yeaman M. R. Lin L. Fu Y. Avanesian V. Bayer A. S. Filler S. G. Lipke P. **& other authors (**2008**).** The antifungal vaccine derived from the recombinant N terminus of Als3p protects mice against the bacterium *Staphylococcus aureus*. Infect Immun 76, 4574–4580 10.1128/IAI.00700-08 18644876 PMC2546811

[r50] Stevens D. A. Clemons K. V. Liu M. **(**2011**).** Developing a vaccine against aspergillosis. Med Mycol 49 (Suppl. 1), S170–S176 10.3109/13693786.2010.497775 20608783

[r51] Tarcha E. J. Basrur V. Hung C. Y. Gardner M. J. Cole G. T. **(**2006**).** Multivalent recombinant protein vaccine against coccidioidomycosis. Infect Immun 74, 5802–5813 10.1128/IAI.00961-06 16988258 PMC1594896

[r52] Tewari R. P. Sharma D. K. Mathur A. **(**1978**).** Significance of thymus-derived lymphocytes in immunity elicited by immunization with ribosomes or live yeast cells of *Histoplasma capsulatum*. J Infect Dis 138, 605–613 10.1093/infdis/138.5.605 309495

[r53] Torosantucci A. Bromuro C. Chiani P. De Bernardis F. Berti F. Galli C. Norelli F. Bellucci C. Polonelli L. **& other authors (**2005**).** A novel glyco-conjugate vaccine against fungal pathogens. J Exp Med 202, 597–606 10.1084/jem.20050749 16147975 PMC2212864

[r54] Wüthrich M. Krajaejun T. Shearn-Bochsler V. Bass C. Filutowicz H. I. Legendre A. M. Klein B. S. **(**2011a**).** Safety, tolerability, and immunogenicity of a recombinant, genetically engineered, live-attenuated vaccine against canine blastomycosis. Clin Vaccine Immunol 18, 783–789 10.1128/CVI.00560-10 21367980 PMC3122516

[r55] Wüthrich M. Gern B. Hung C. Y. Ersland K. Rocco N. Pick-Jacobs J. Galles K. Filutowicz H. Warner T. **& other authors (**2011b**).** Vaccine-induced protection against 3 systemic mycoses endemic to North America requires Th17 cells in mice. J Clin Invest 121, 554–568 10.1172/JCI43984 21206087 PMC3026727

[r56] Wyckoff E. E. Pishko E. J. Kirkland T. N. Cole G. T. **(**1995**).** Cloning and expression of a gene encoding a T-cell reactive protein from *Coccidioides immitis*: homology to 4-hydroxyphenylpyruvate dioxygenase and the mammalian F antigen. Gene 161, 107–111 10.1016/0378-1119(95)00250-A 7642122

[r57] Xin H. Cutler J. E. **(**2011**).** Vaccine and monoclonal antibody that enhance mouse resistance to candidiasis. Clin Vaccine Immunol 18, 1656–1667 10.1128/CVI.05215-11 21832099 PMC3187024

[r58] Xin H. Dziadek S. Bundle D. R. Cutler J. E. **(**2008**).** Synthetic glycopeptide vaccines combining β-mannan and peptide epitopes induce protection against candidiasis. Proc Natl Acad Sci U S A 105, 13526–13531 10.1073/pnas.0803195105 18725625 PMC2533223

[r59] Xue J. Chen X. Selby D. Hung C. Y. Yu J. J. Cole G. T. **(**2009**).** A genetically engineered live attenuated vaccine of *Coccidioides posadasii* protects BALB/c mice against coccidioidomycosis. Infect Immun 77, 3196–3208 10.1128/IAI.00459-09 19487479 PMC2715678

[r60] Zelante T. De Luca A. Bonifazi P. Montagnoli C. Bozza S. Moretti S. Belladonna M. L. Vacca C. Conte C. **& other authors (**2007**).** IL-23 and the Th17 pathway promote inflammation and impair antifungal immune resistance. Eur J Immunol 37, 2695–2706 10.1002/eji.200737409 17899546

